# Supplementary material for: ATAC-Seq Identifies Chromatin Landscapes Linked to the Regulation of Oxidative Stress in the Human Fungal Pathogen Candida albicans
Source: J Fungi (Basel). 2020 Sep 21;6(3):182. doi: 10.3390/jof6030182 (PMC7559329; doi:10.3390/jof6030182)
Supplement: Supplementary file 1 [file jof-06-00182-s001.zip › jof-930743-Suppl_Materials_JoF_R1.pdf]

Supplementary materials for

ATAC-seq identifies chromatin landscapes linked to the regulation of oxidative stress in the human fungal pathogen *Candida albicans*

Sabrina Jenull[1,*], Michael Tscherner[1,*], Theresia Mair[1] and Karl Kuchler[1,#]

From the
[1]Department of Medical Biochemistry, Max Perutz Labs Vienna, Medical University of Vienna, Campus Vienna Biocenter, Dr. Bohr-Gasse 9/2, A-1030 Vienna

Running title: ATACing fungal chromatin
* contributed equally
# To whom correspondence should be addressed
e-mail: karl.kuchler@meduniwien.ac.at

Figures:
Figure S1: Number of mapped ATAC-seq reads to each *C. albicans* chromosome.
Figure S2: Analysis of genes with decreased chromatin accessibility in regions upstream their TSS.
Figure S3: PCA of detected nucleosome-free ATAC-seq peaks.

Tables:
Table S1: Nextera primers used for ATAC-seq library preparation
Table S2: *K*-means clustering result and transcriptional regulation of associated genes
Table S3: MACS2 peak calling and edgeR analysis result
Table S4: Merged ATAC-seq and RNA-seq result
Table S5: Peaks upstream of genes with altered chromatin accessibility upon oxidative stress without changes in gene expression

Figure S1

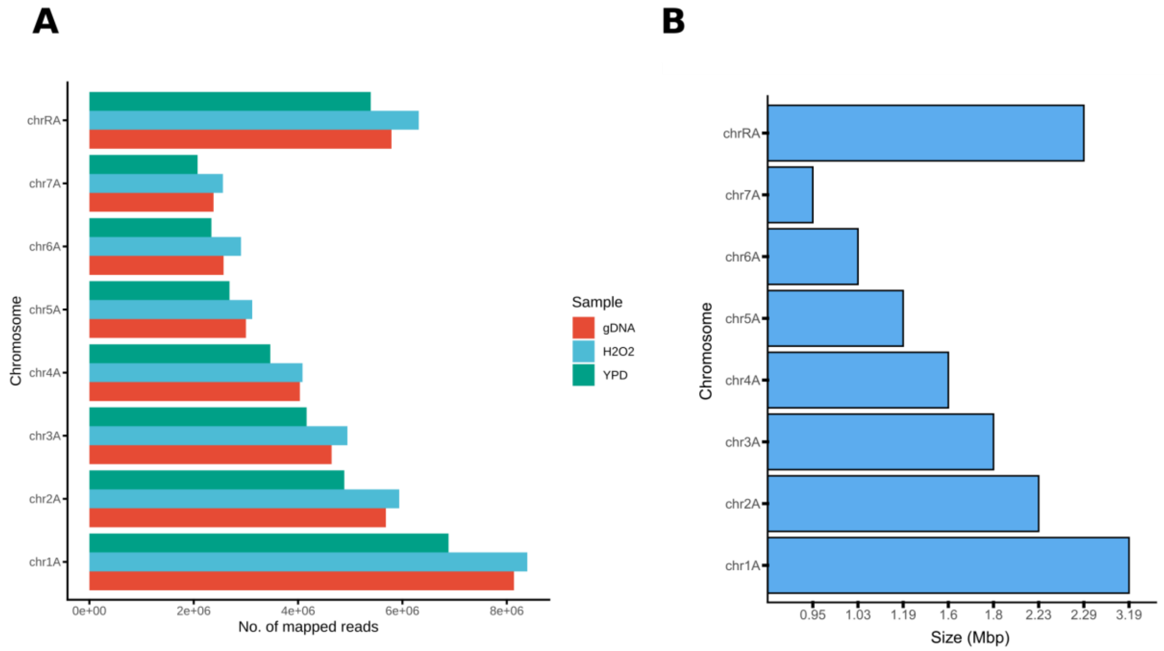

Figure S1. Number of mapped ATAC-seq reads to each *C. albicans* chromosome.

A-B) The aligned reads from each biological replicate of the gDNA, H_2O_2-treated (H_2O_2) and non-treated (YPD) ATAC-seq libraries were merged and the number of mapped reads (x-axis) per chromosome (y-axis) was plotted (A). As comparison, the total size (in Mbp) of each *C. albicans* chromosome is presented as well (B).

Figure S2

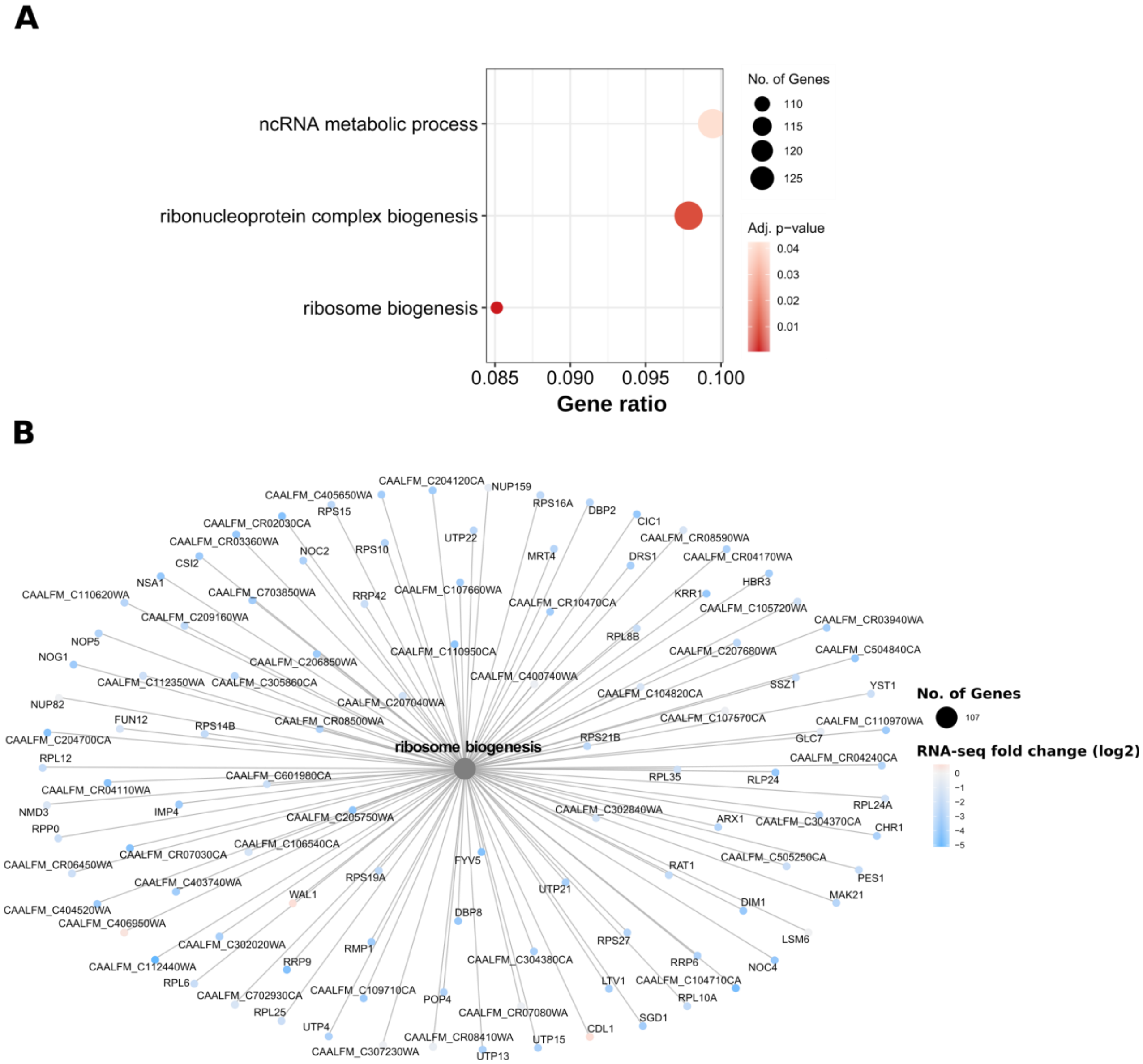

Figure S2. Analysis of genes with decreased chromatin accessibility in regions upstream their TSS.
A-B) Genomic regions from cluster 4 (see Figure 2C-D) were annotated to the next downstream gene and subjected to GO term enrichment analysis. Enriched biological processes are represented as dotplot (A) and cnet plot for the biological process "ribosome biogenesis" (B). The cnet plot depicts the genes associated with the presented GO term and the log2-fold change from RNA-seq data overlaid as color gradient. See figure legend from Figure 2E for detailed description.

Figure S3

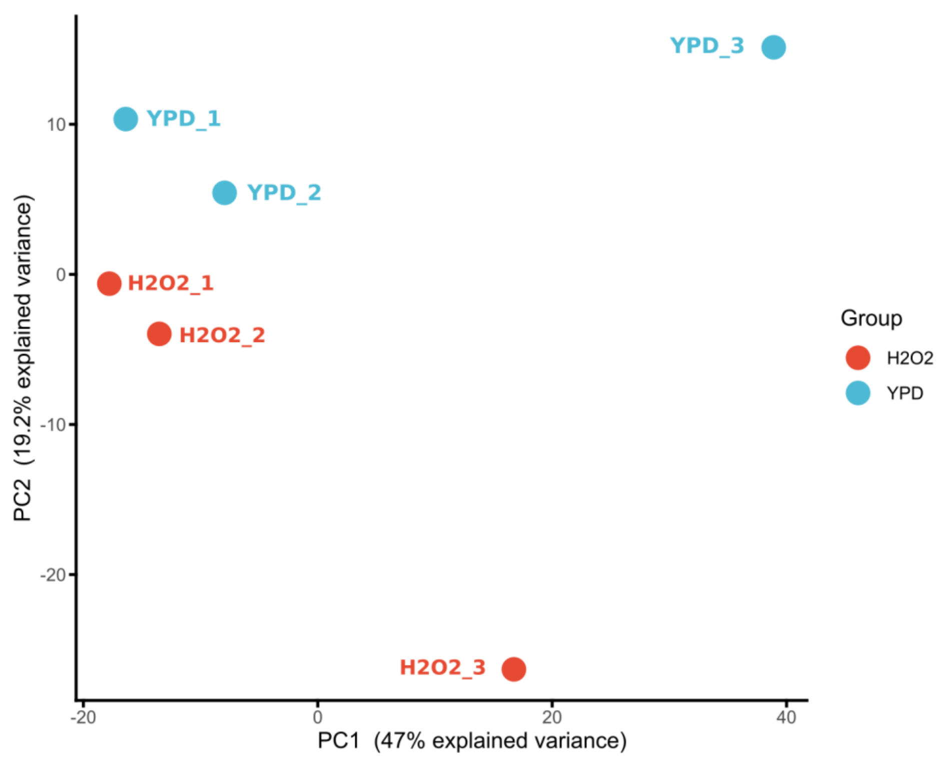

Figure S3. PCA of detected nucleosome-free ATAC-seq peaks.

Called nucleosome-free ATAC-seq peaks from all samples were merged and subjected to PCA based on the presence or absence of ATAC-seq peaks in each replicate and condition (YPD and H_2O_2). Each dot in blue represents three biological replicates from YPD-grown cells (YPD) and each dot in red represents three biological replicates of H_2O_2-treated samples (H_2O_2). The x-axis shows the principal component 1 (PC1) and the y-axis the principal component 2 (PC2).

Table S1. Nextera primers used for ATAC-seq library preparation.

name	barcode	sequence (5' -> 3')
Ad1_noMX		AATGATACGGCGACCACCGAGATCTACACTCGTCGGCAGCGTCAGATGTG
Ad2.30_TTGCTAAG	TTGCTAAG	CAAGCAGAAGACGGCATACGAGATCTTAGCAAGTCTCGTGGGCTCGGAGATGT
Ad2.31_ATAAGTTA	ATAAGTTA	CAAGCAGAAGACGGCATACGAGATTAACTTATGTCTCGTGGGCTCGGAGATGT
Ad2.32_ATCACTCG	ATCACTCG	CAAGCAGAAGACGGCATACGAGATCGAGTGATGTCTCGTGGGCTCGGAGATGT
Ad2.33_GTTAACAG	GTTAACAG	CAAGCAGAAGACGGCATACGAGATCTGTTAACGTCTCGTGGGCTCGGAGATGT
Ad2.38_GAAATGCC	GAAATGCC	CAAGCAGAAGACGGCATACGAGATGGCATTTCGTCTCGTGGGCTCGGAGATGT
Ad2.39_AACGCCAT	AACGCCAT	CAAGCAGAAGACGGCATACGAGATATGGCGTTGTCTCGTGGGCTCGGAGATGT
