# Supplementary figures and images for: ATAC-Seq Identifies Chromatin Landscapes Linked to the Regulation of Oxidative Stress in the Human Fungal Pathogen Candida albicans
Source: J Fungi (Basel). 2020 Sep 21;6(3):182. doi: 10.3390/jof6030182 (PMC7559329; doi:10.3390/jof6030182)

Figure S1

A

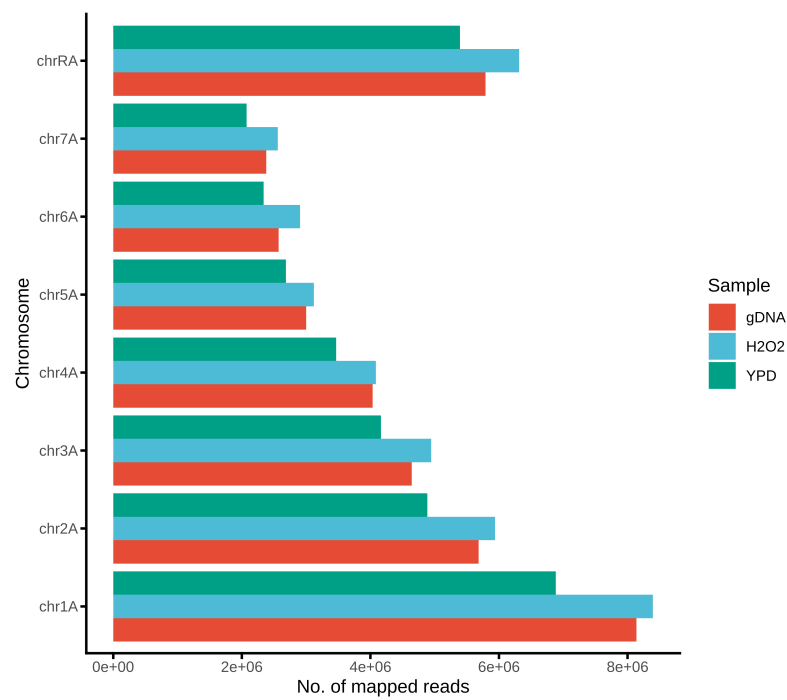

B

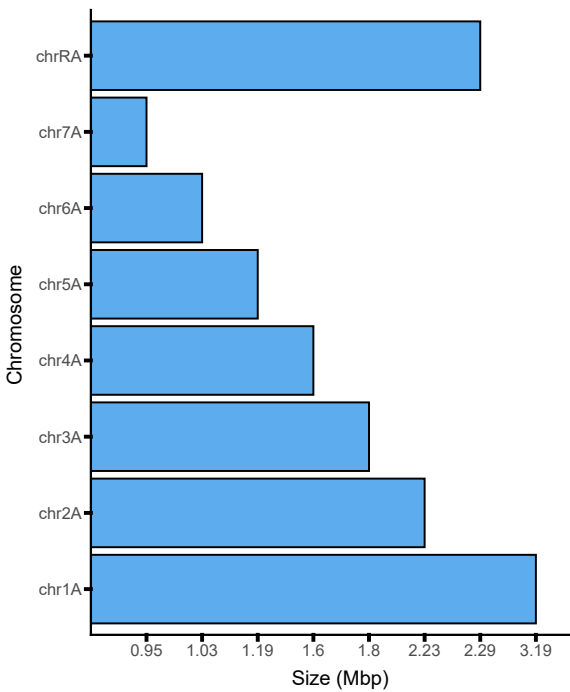

Supplement: Supplementary file 1 [file jof-06-00182-s001.zip › FigureS1.pdf]

**A**

# B

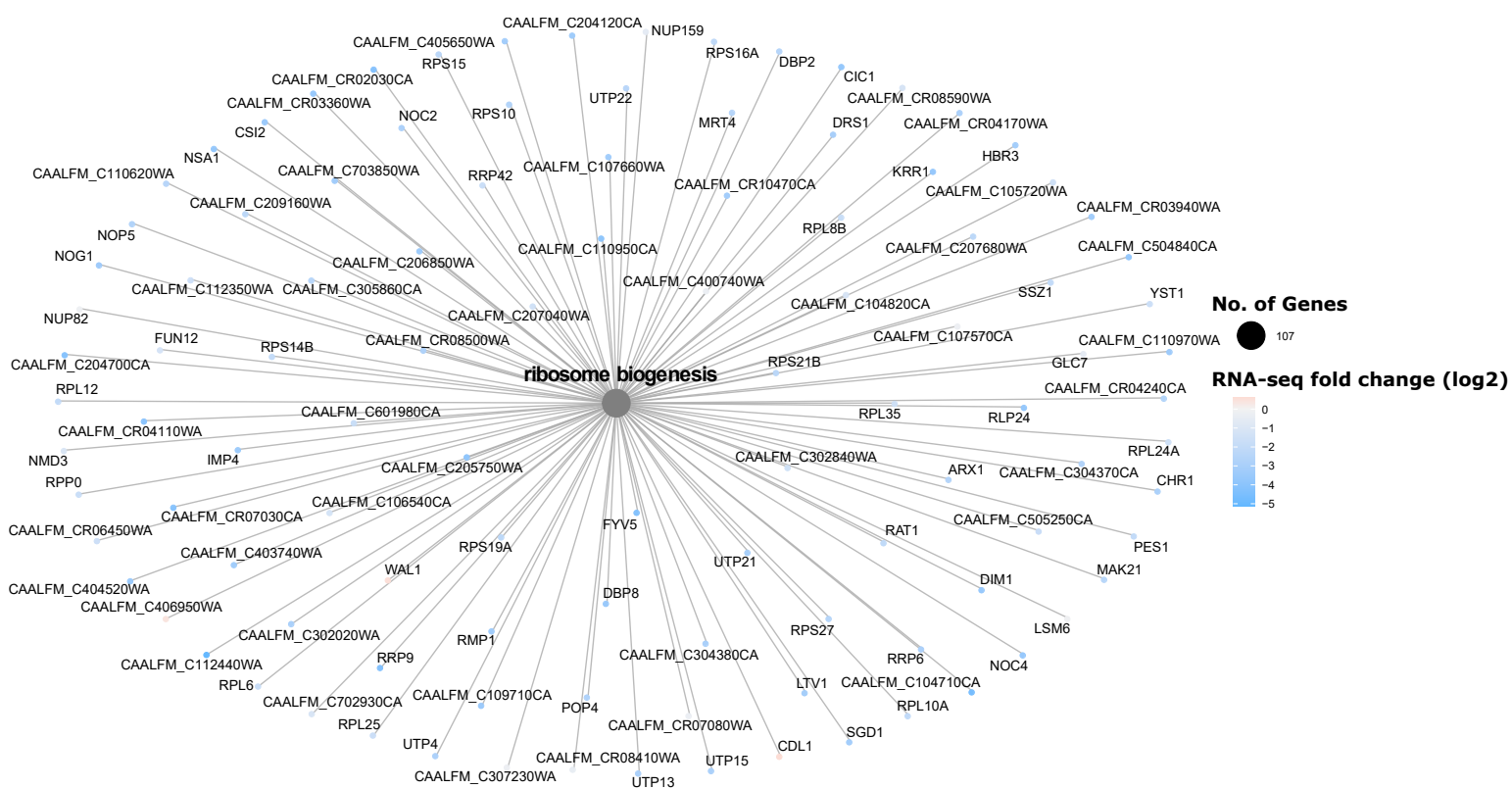

Supplement: Supplementary file 1 [file jof-06-00182-s001.zip › FigureS2.pdf]

# Figure S3

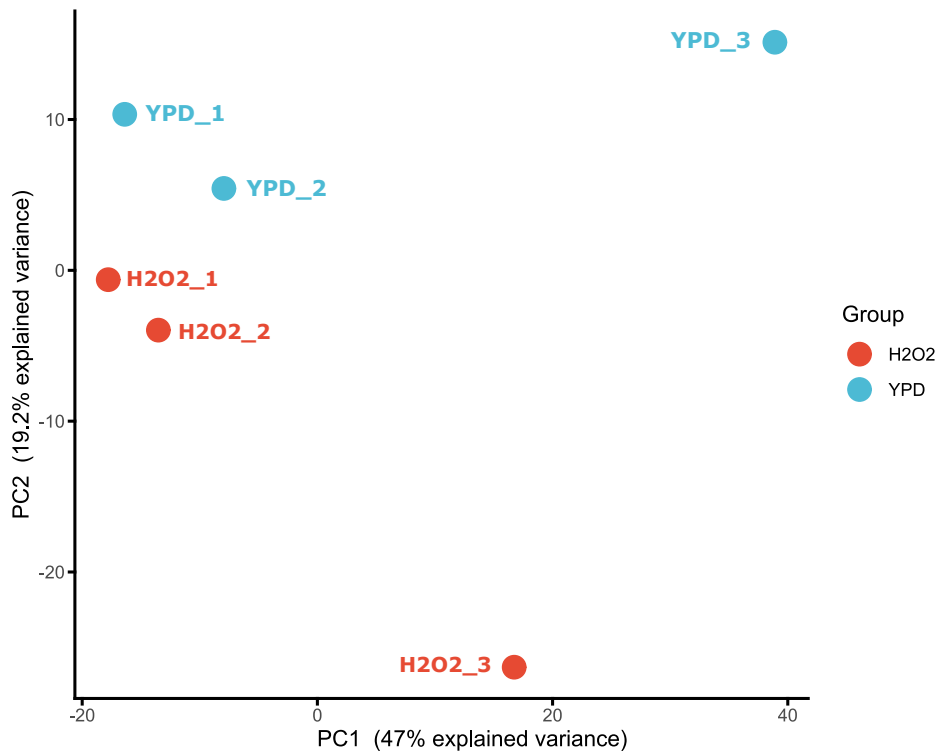

Supplement: Supplementary file 1 [file jof-06-00182-s001.zip › FigureS3.pdf]
